# Supplementary material for: Weight loss during follow-up in patients with acute heart failure: From the KCHF registry
Source: PLoS One. 2023 Jun 23;18(6):e0287637. doi: 10.1371/journal.pone.0287637 (PMC10289349; doi:10.1371/journal.pone.0287637)

S2 Fig. Study patients flow in the Sensitivity analysis. (≥10% decrease in body weight, -10%< body weight change ≤ -5% and no weight loss [-5%< body weight change])

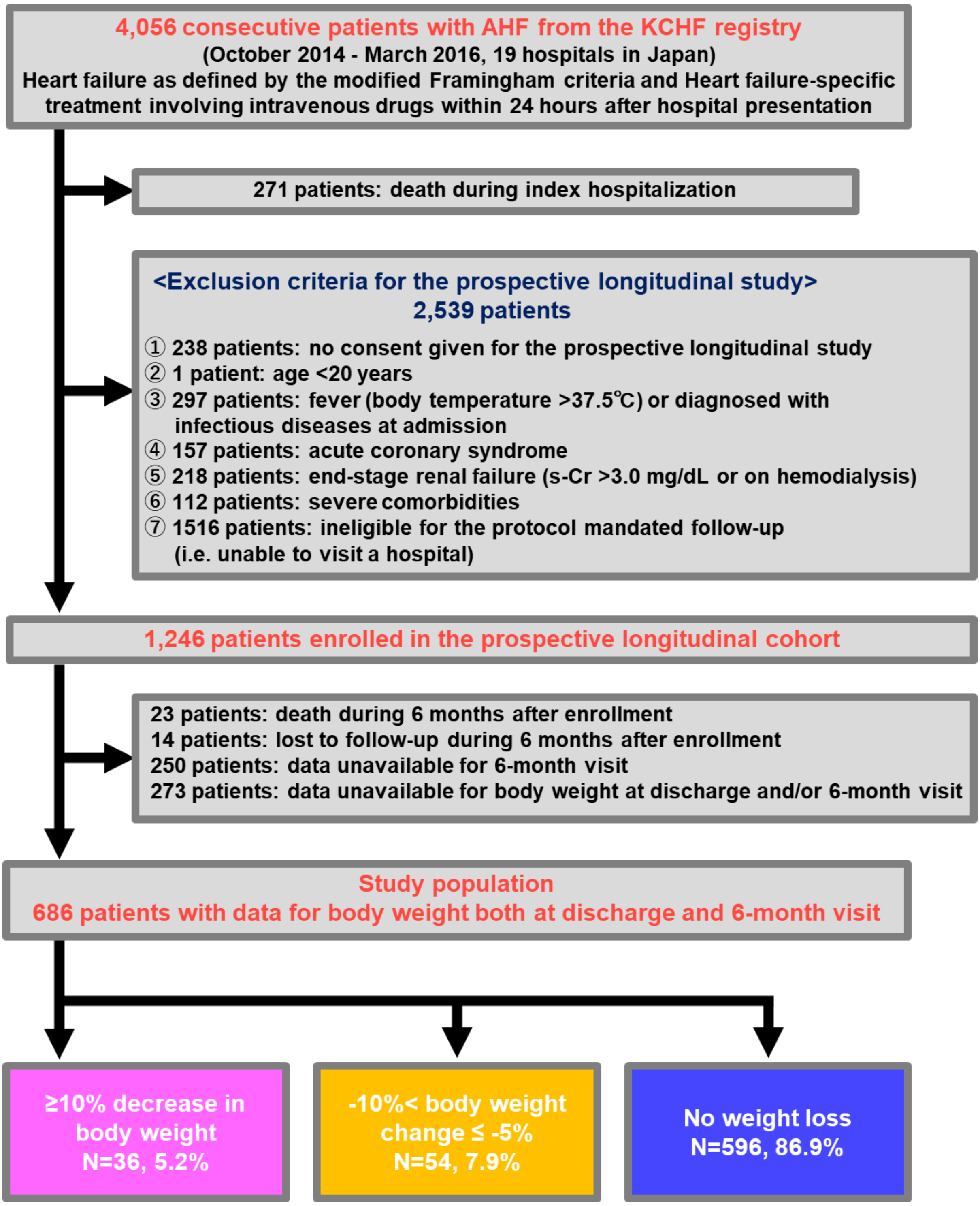

Supplement: S2 Fig — AHF, acute heart failure; KCHF, Kyoto Congestive Heart Failure; s-Cr, serum creatinine. (PDF) [file pone.0287637.s002.pdf]
