# Supplementary material for: Weight loss during follow-up in patients with acute heart failure: From the KCHF registry
Source: PLoS One. 2023 Jun 23;18(6):e0287637. doi: 10.1371/journal.pone.0287637 (PMC10289349; doi:10.1371/journal.pone.0287637)
Supplement: S3 Fig — CI, Confidence interval; HR, Hazard ratio. (PDF) [file pone.0287637.s003.pdf]

S3 Fig. Kaplan-Meier curves in the Sensitivity analysis. (weight loss, no weight change and weight gain)

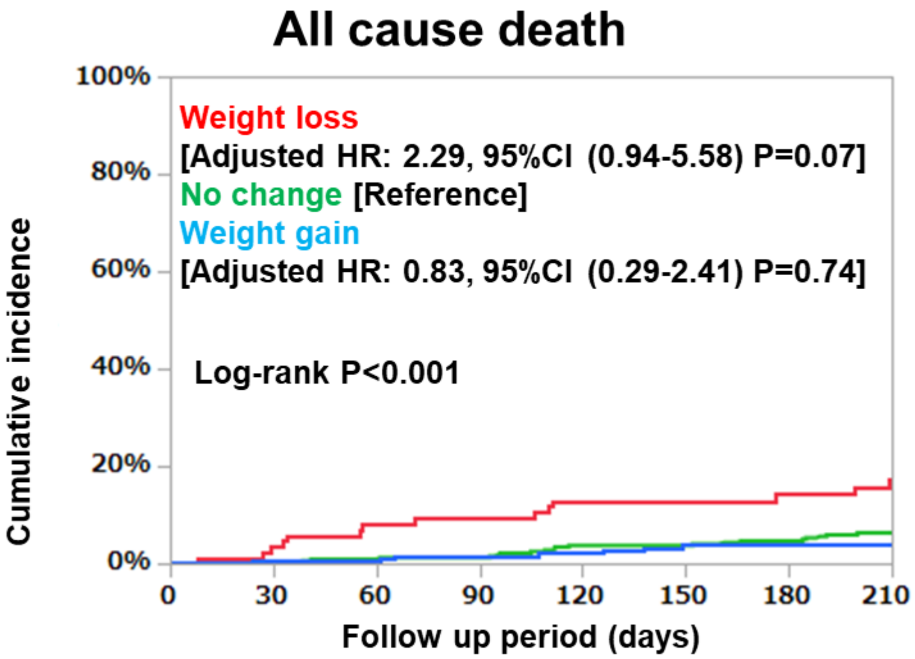

Interval (days)	0	60	120	180
**Weight loss**				
N of patients with event		7	11	12
N of patients at risk	90	78	74	66
Cumulative incidence		8.2%	12.9%	14.2%
**No change**				
N of patients with event		3	12	15
N of patients at risk	402	326	315	294
Cumulative incidence		0.9%	3.7%	4.6%
**Weight gain**				
N of patients with event		1	4	7
N of patients at risk	194	181	177	163
Cumulative incidence		0.5%	2.2%	3.9%
