# Supplementary material for: Weight loss during follow-up in patients with acute heart failure: From the KCHF registry
Source: PLoS One. 2023 Jun 23;18(6):e0287637. doi: 10.1371/journal.pone.0287637 (PMC10289349; doi:10.1371/journal.pone.0287637)
Supplement: S4 Fig — CI, Confidence interval; HR, Hazard ratio. (PDF) [file pone.0287637.s004.pdf]

S4 Fig. Kaplan-Meier curves in the Sensitivity analysis. (≥10% decrease in body weight, -10%< body weight change ≤ -5% and no weight loss [-5%< body weight change])

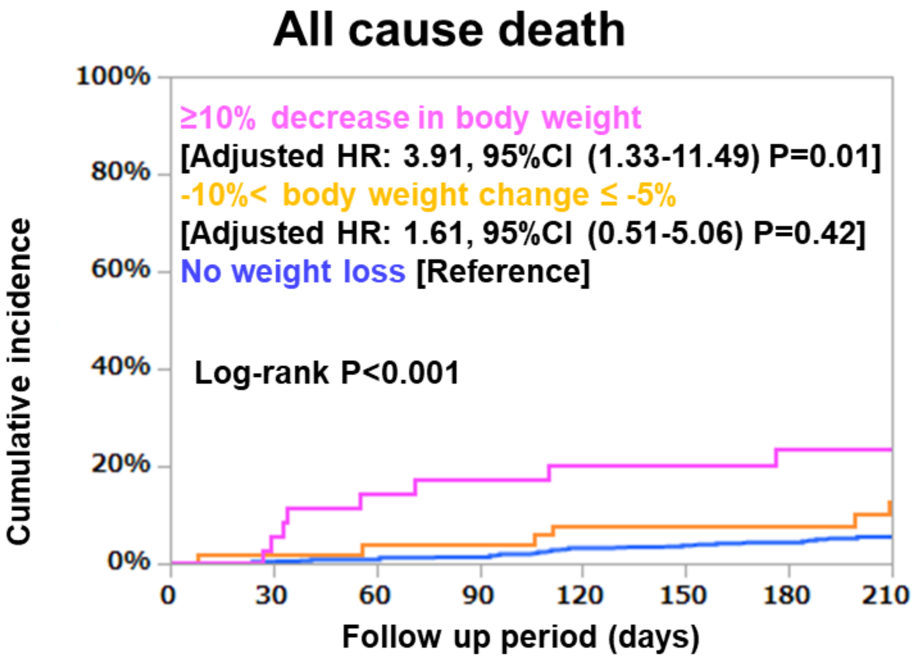

Interval (days)	0	60	120	180
**≥10% decrease in body weight**				
N of patients with event		5	7	8
N of patients at risk	36	29	27	23
Cumulative incidence		14.4%	20.3%	23.6%
**-10%< body weight change ≤ -5%**				
N of patients with event		2	4	4
N of patients at risk	54	49	47	43
Cumulative incidence		3.9%	7.8%	7.8%
**No weight loss**				
N of patients with event		4	16	22
N of patients at risk	596	507	492	457
Cumulative incidence		0.8%	3.1%	4.3%
