# Supplementary figures and images for: Weight loss during follow-up in patients with acute heart failure: From the KCHF registry
Source: PLoS One. 2023 Jun 23;18(6):e0287637. doi: 10.1371/journal.pone.0287637 (PMC10289349; doi:10.1371/journal.pone.0287637)

**S5 Fig. Subgroup analysis**

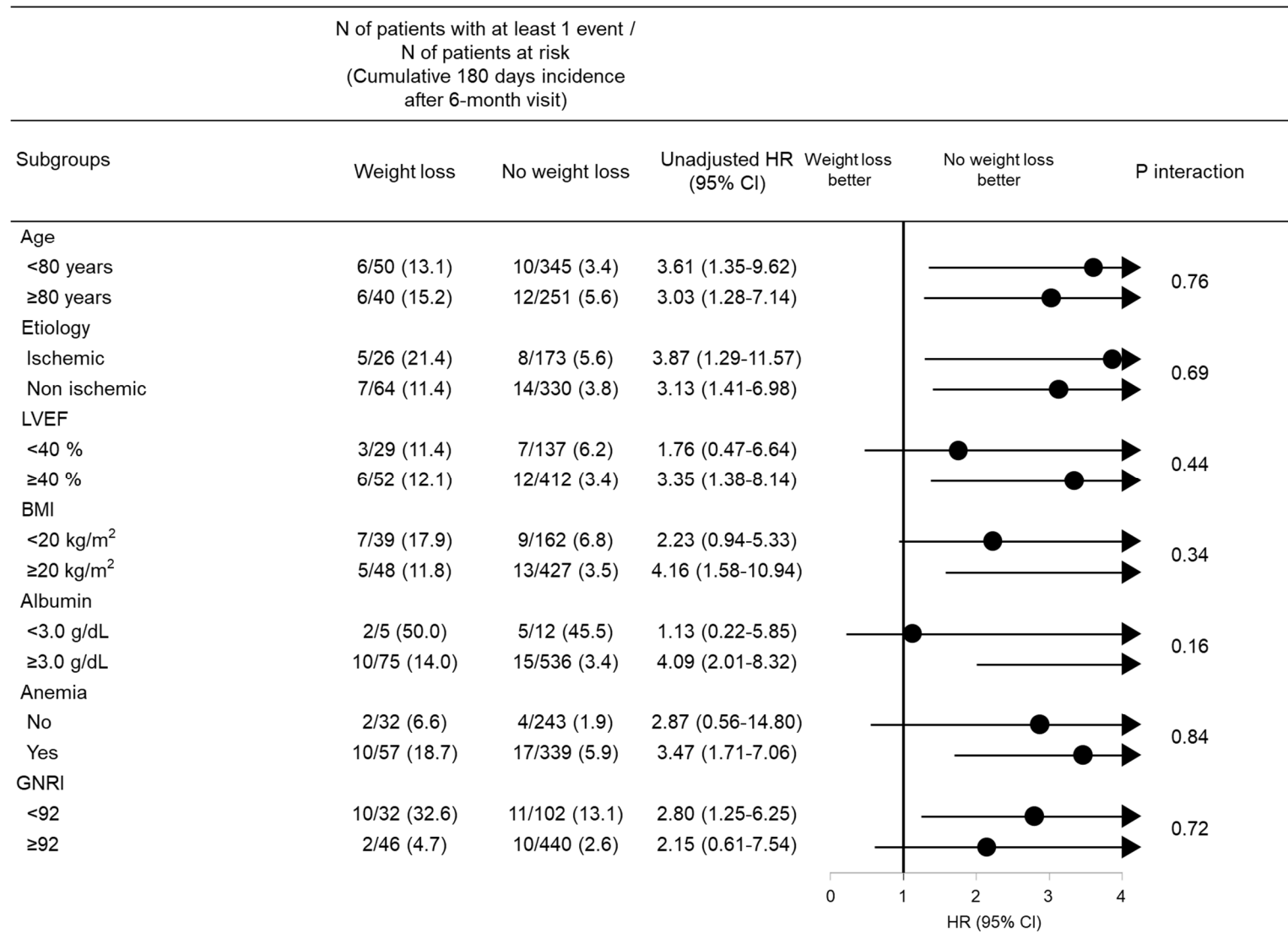

Supplement: S5 Fig — BMI, body mass index; CI, confidence interval; GNRI, geriatric nutritional risk index; HR, hazard ratio; LVEF, left ventricular ejection fraction. (PDF) [file pone.0287637.s005.pdf]
