# Supplementary material for: Weight loss during follow-up in patients with acute heart failure: From the KCHF registry
Source: PLoS One. 2023 Jun 23;18(6):e0287637. doi: 10.1371/journal.pone.0287637 (PMC10289349; doi:10.1371/journal.pone.0287637)
Supplement: S1 Table — Values are number (%), mean ± standard deviation (SD), or median (interquartile range). P values were calculated using the chi square test for categorical variables, and the Student’s t test or Wilcoxon rank sum test for continuous variables. ACEI, angiotensin-converting enzyme inhibitor; ARB, angiotensin-receptor blocker; BMI, body mass index; MRA, mineralocorticoid receptor antagonist. (PDF) [file pone.0287637.s006.pdf]

S1 Table: Baseline characteristics of the patients available with body weight data and those unavailable with body weight data at discharge

Variable	Available BW change data (N=686)	Unavailable BW change data (N=560)
**Clinical Characteristic**		
Age, years	78 (70-84)	79 (71-85)
Age≥80 years	291 (42.4)	271 (48.4)
Men	405 (59.0)	317 (56.6)
Body weight at discharge, kg	55.5 ± 14.6	52.7 ± 12.9
Hypertension	507 (73.9)	410 (73.2)
Diabetes	258 (37.6)	220 (39.3)
Atrial fibrillation or flutter	376 (54.8)	325 (58.0)
Previous myocardial infarction	171 (24.9)	112 (20.0)
Malignancy	102 (14.9)	81 (14.5)
LVEF, %	50.6 ± 16.1	48.7 ± 16.4
HFrEF (LVEF<40%)	166 (26.3)	67 (34.2)
eGFR<30 mL/min/1.73m^2	161 (23.9)	56 (25.0)
Albumin <3.0 g/dL	17 (2.7)	15 (7.5)
Anemia	396 (59.0)	138 (62.2)
ACEI or ARB at discharge	337 (58.9)	115 (53.2)
β blocker at discharge	440 (76.8)	140 (64.2)
MRA	266 (46.7)	87 (39.9)

Values are number (%), mean ± SD, or median (interquartile range). P values were calculated using the chi square test for categorical variables, and the Student's t test or Wilcoxon rank sum test for continuous variables.

ACEI, angiotensin-converting enzyme inhibitor; ARB, angiotensin-receptor blocker; BMI, body mass index; MRA, mineralocorticoid receptor antagonist
