# Supplementary material for: Weight loss during follow-up in patients with acute heart failure: From the KCHF registry
Source: PLoS One. 2023 Jun 23;18(6):e0287637. doi: 10.1371/journal.pone.0287637 (PMC10289349; doi:10.1371/journal.pone.0287637)
Supplement: S2 Table — Values are number (%), mean ± standard deviation (SD), or median (interquartile range). P values were calculated using the chi square test for categorical variables, and the Student’s t test or Wilcoxon rank sum test for continuous variables. ACEI, angiotensin-converting enzyme inhibitor; ARB, angiotensin-receptor blocker; BMI, body mass index; BP, blood pressure; BNP, brain-type natriuretic peptide; eGFR, estimated glomerular filtration rate; HFrEF, heart failure with reduced ejection fraction; LVEF, left ventricular ejection fraction; MRA, mineralocorticoid receptor antagonist; NT-proBNP, N-terminal pro-brain-type natriuretic peptide; NYHA, New York Heart Association. (PDF) [file pone.0287637.s007.pdf]

S2 Table: Baseline characteristics at discharge

	Total (n=686)	Weight loss (n=90)	No weight loss (n=596)	P value	Evaluable N
**Clinical Characteristic**					
Age, years	78 (70-84)	79 (72-84)	77 (69-84)	0.27	686
Age≥80 years	291 (42.4)	40 (44.4)	251 (42.1)	0.68	686
Men	405 (59.0)	50 (55.6)	355 (59.6)	0.47	686
Body weight at discharge, kg	55.5 ± 14.6	59.3 ± 19.4	54.9 ± 13.7	0.009	686
BMI at discharge	22.3 ± 4.7	24.0 ± 6.9	22.0 ± 4.3	<0.001	676
BMI<20 at discharge	230 (34.0)	24 (27.6)	206 (35.0)	0.17	676
**Medical history**					
Hypertension	507 (73.9)	71 (78.9)	436 (73.2)	0.25	686
Diabetes	258 (37.6)	36 (40.0)	222 (37.2)	0.62	686
Dyslipidemia	294 (42.9)	41 (45.6)	253 (42.5)	0.58	686
Atrial fibrillation or flutter	376 (54.8)	49 (54.4)	327 (54.9)	0.94	686
Previous myocardial infarction	171 (24.9)	24 (26.7)	147 (24.7)	0.68	686
Previous stroke	113 (16.5)	16 (17.8)	97 (16.3)	0.72	686
Chronic kidney disease	304 (44.3)	48 (53.3)	256 (43.0)	0.06	686
Chronic lung disease	88 (12.8)	11 (12.2)	77 (12.9)	0.85	686
Malignancy*	102 (14.9)	19 (21.1)	83 (13.9)	0.07	686
Cognitive dysfunction	73 (10.6)	10 (11.1)	63 (10.6)	0.88	686
**Vital signs at discharge**					
Heart rate, bpm	70.6 ± 12.5	71.6 ± 13.8	70.4 ± 12.4	0.39	684
Systolic BP, mmHg	113.9 ± 16.7	117.1 ± 16.7	113.5 ± 16.7	0.055	686
Diastolic BP, mmHg	64.5 ± 11.7	66.7 ± 12.8	64.2 ± 11.5	0.06	686
**Test at discharge**					
LVEF, %	45.0 ± 16.8	46.9 ± 17.4	44.7 ± 16.7	0.24	683
HFrEF (LVEF<40%)	272 (39.7)	34 (37.8)	238 (39.9)	0.70	686
BNP, pg/ml	232.0 (115.5-459.8)	236.4 (134.0-582.9)	230.7 (111.1-442.5)	0.18	450
NT-proBNP, pg/ml	1636 (667-3337)	2165 (518-2798)	1571 (678-3360)	0.71	158
Serum creatinine, mg/dl	1.11 (0.85-1.44)	1.13 (0.87-1.53)	1.11 (0.85-1.42)	0.34	681

eGFR, ml/min/1.73m^2	47.6 ± 20.4	44.7 ± 19.3	48.0 ± 20.5	0.15	681
<30 ml/min/1.73m^2	127 (18.6)	23 (25.8)	104 (17.6)	0.06	681
Albumin, g/dl	3.51 ± 0.44	3.42 ± 0.50	3.52 ± 0.43	0.06	632
<3.0 g/dl	67 (10.6)	15 (18.3)	52 (9.5)	0.02	632
Sodium, mEq/l	138.7 ± 3.1	139.0 ± 3.1	138.7 ± 3.1	0.38	678
<135 mEq/l	59 (8.7)	8 (9.1)	51 (8.6)	0.89	678
Hemoglobin, g/dl	12.1 ± 2.3	11.8 ± 2.3	12.1 ± 2.3	0.24	670
Anemia	399 (59.6)	53 (60.2)	346 (59.5)	0.89	670
**Medication at discharge**					
ACEIs or ARBs	465 (67.8)	66 (73.3)	399 (66.9)	0.23	686
β blockers	529 (77.1)	75 (83.3)	454 (76.2)	0.13	686
MRAs	354 (51.6)	49 (54.4)	305 (51.2)	0.56	686
Diuretics	601 (87.6)	83 (92.2)	518 (86.9)	0.15	686
**Presence of edema at discharge**	76 (11.2)	18 (20.9)	58 (9.8)	0.002	676

Values are number (%), mean ± standard deviation (SD), or median (interquartile range). P values were calculated using the chi square test for categorical variables, and the Student's t test or Wilcoxon rank sum test for continuous variables.

ACEI, angiotensin-converting enzyme inhibitor; ARB, angiotensin-receptor blocker; BMI, body mass index; BP, blood pressure; BNP, brain-type natriuretic peptide; eGFR, estimated glomerular filtration rate; HFrEF, heart failure with reduced ejection fraction; LVEF, left ventricular ejection fraction; MRA, mineralocorticoid receptor antagonist; NT-proBNP, N-terminal pro-brain-type natriuretic peptide; NYHA, New York Heart Association.
