# Supplementary material for: Weight loss during follow-up in patients with acute heart failure: From the KCHF registry
Source: PLoS One. 2023 Jun 23;18(6):e0287637. doi: 10.1371/journal.pone.0287637 (PMC10289349; doi:10.1371/journal.pone.0287637)
Supplement: S4 Table — Values are number (%), mean ± standard deviation (SD), or median (interquartile range). P values were calculated using the chi square test for categorical variables, and 1-way ANOVA or Kruskal-Wallis test for continuous variables. The changes (delta, Δ) were calculated according to the following equation: (the value at 6-month visit)—(the value at discharge). ACEI, angiotensin-converting enzyme inhibitor; ARB, angiotensin-receptor blocker; BMI, body mass index; BP, blood pressure; BNP, brain-type natriuretic peptide; eGFR, estimated glomerular filtration rate; GNRI, geriatric nutritional risk index; HFrEF, heart failure with reduced ejection fraction; LVEF, left ventricular ejection fraction; MRA, mineralocorticoid receptor antagonist; NT-proBNP, N-terminal pro-brain-type natriuretic peptide; NYHA, New York Heart Association. * Risk-adjusting variables selected for the Cox proportional hazard models and the Fine-Gray subdistribution hazard model. (PDF) [file pone.0287637.s009.pdf]

S4 Table: Baseline characteristics in sensitivity analysis (≥10% decrease in body weight, -10%< body weight change ≤ -5% and no weight loss[-5%< body weight change])

	Total (N=686)	≥10% decrease in body weight (N=36)	-10%< body weight change ≤ -5% (N=54)	No weight loss (N=596)	P value	Evaluable N
**Clinical Characteristic**						
Age, years	78 (70-84)	79 (75-84)	78 (70-85)	77 (69-84)	0.45	686
Age≥80 years*	291 (42.4)	16 (44.4)	24 (44.4)	251 (42.1)	0.92	686
Men*	405 (59.0)	20 (55.6)	30 (55.6)	355 (59.6)	0.77	686
Body weight at discharge, kg	55.5 ± 14.6	61.3 ± 23.7	57.9 ± 15.9	54.9 ± 13.7	0.02	686
Body weight at 6 month visit, kg	56.5 ± 14.8	52.6 ± 18.6	53.7 ± 14.9	57.0 ± 14.5	0.08	686
⊿Body weight	1.0 ± 4.8	-8.7 ± 6.3	-4.2 ± 1.3	2.1 ± 3.8	<0.001	686
BMI at discharge	22.3 ± 4.7	24.9 ± 8.6	23.5 ± 5.5	22.0 ± 4.3	<0.001	676
BMI at 6 month visit	22.7 ± 4.7	21.3 ± 6.6	21.8 ± 5.1	22.8 ± 4.5	0.07	676
BMI<20 at 6 month visit*	201 (29.7)	18 (51.4)	21 (40.4)	162 (27.5)	0.002	676
**Etiology**						
Ischemic	199 (29.0)	10 (27.8)	16 (29.6)	173 (29.0)	0.98	
**Medical history**						
Hypertension	507 (73.9)	32 (88.9)	39 (72.2)	436 (73.2)	0.11	686
Diabetes	258 (37.6)	15 (41.7)	21 (38.9)	222 (37.2)	0.85	686
Dyslipidemia	294 (42.9)	16 (44.4)	25 (46.3)	253 (42.5)	0.84	686
Atrial fibrillation or flutter	376 (54.8)	20 (55.6)	29 (53.7)	327 (54.9)	0.98	686
Previous myocardial infarction	171 (24.9)	6 (16.7)	18 (33.3)	147 (24.7)	0.19	686
Previous stroke	113 (16.5)	10 (27.8)	6 (11.1)	97 (16.3)	0.11	686
Chronic kidney disease	304 (44.3)	19 (52.8)	29 (53.7)	256 (43.0)	0.18	686
Chronic lung disease	88 (12.8)	6 (16.7)	5 (9.3)	77 (12.9)	0.58	686
Malignancy*	102 (14.9)	8 (22.2)	11 (20.4)	83 (13.9)	0.20	686
Cognitive dysfunction	73 (10.6)	7 (19.4)	3 (5.6)	63 (10.6)	0.11	686
**Vital signs at 6 month visit**						
Heart rate, bpm	74.7 ± 13.8	74.8 ± 17.6	77.1 ± 13.7	74.4 ± 13.5	0.41	653
Systolic BP, mmHg	121.2 ± 21.6	115.7 ± 29.4	114.0 ± 25.1	122.2 ± 20.5	0.001	661

Diastolic BP, mmHg	67.7 ± 13.5	66.4 ± 16.7	65.6 ± 13.8	68.0 ± 13.2	0.39	660
NYHA class III or IV	32 (7.0)	3 (16.7)	3 (7.9)	26 (6.5)	0.25	458
**Test at 6 month visit**						
LVEF, %	50.6 ± 16.1	51.8 ± 17.4	48.4 ± 18.0	50.8 ± 15.8	0.54	630
HFrEF (LVEF<40%)*	166 (26.3)	8 (27.6)	21 (40.4)	137 (25.0)	0.054	630
⊿LVEF, %	6.1 ± 13.4	2.3 ± 10.9	3.0 ± 13.1	6.6 ± 13.5	0.055	627
BNP, pg/ml	181 (78-382)	236 (114-580)	241 (96-419)	173 (73-365)	0.15	527
⊿BNP, pg/ml	-26 ± 308	36 ± 420	-74 ± 302	-24 ± 302	0.48	436
NT-proBNP, pg/ml	1156 (545-2611)	1418 (738-12506)	618 (219-2013)	1181 (559-2629)	0.07	267
Serum creatinine, mg/dl	1.14 (0.89-1.57)	1.21 (0.81-1.66)	1.22 (0.91-1.73)	1.14 (0.90-1.54)	0.53	673
⊿creatinine, mg/dl	0.09 ± 0.41	0.07 ± 0.36	0.02 ± 0.44	0.09 ± 0.41	0.44	668
eGFR, ml/min/1.73m^2	45.3 ± 20.6	45.5 ± 20.1	41.4 ± 18.1	45.6 ± 20.8	0.37	673
<30 ml/min/1.73m^2*	161 (23.9)	9 (25.7)	13 (24.5)	139 (23.8)	0.96	673
⊿eGFR, ml/min/1.73m^2	-2.3 ± 12.9	-1.8 ± 14.0	-1.1 ± 12.4	-2.4 ± 12.9	0.76	668
Albumin, g/dl	3.91 ± 0.49	3.70 ± 0.59	3.79 ± 0.51	3.94 ± 0.48	0.006	628
<3.0 g/dl*	17 (2.7)	3 (10.0)	2 (4.0)	12 (2,2)	0.03	628
⊿Albumin, g/dl	0.40 ± 0.47	0.31 ± 0.40	0.41 ± 0.44	0.40 ± 0.48	0.61	580
Sodium, mEq/l	139.6 ± 3.3	139.2 ± 3.8	139.4 ± 3.0	139.6 ± 3.3	0.68	669
<135 mEq/l	42 (6.3)	5 (14.3)	2 (3.8)	35 (6.0)	0.11	669
⊿Sodium, mEq/l	0.8 ± 3.5	0.4 ± 2.2	0.3 ± 3.7	0.9 ± 3.6	0.40	661
Hemoglobin, g/dl	12.0 ± 2.1	11.5 ± 2.4	11.5 ± 1.8	12.1 ± 2.2	0.049	671
Anemia*	396 (59.0)	21 (60.0)	36 (66.7)	339 (58.2)	0.48	671
⊿Hemoglobin, g/dl	-0.1 ± 1.9	-0.3 ± 2.1	-0.3 ± 1.6	-0.1 ± 1.9	0.54	655
**Medication at 6 month visit**						
ACEIs or ARBs*	337 (58.9)	16 (55.2)	20 (45.5)	301 (60.3)	0.14	572
β-blockers*	440 (76.8)	25 (83.3)	34 (77.3)	381 (76.4)	0.68	573
MRAs*	266 (46.7)	17 (56.7)	23 (52.3)	226 (45.6)	0.37	570
Diuretics	482 (84.0)	27 (90.0)	39 (88.6)	416 (83.2)	0.42	574
**Nutritional score at 6 month visit**						
GNRI	101.4 ± 12.7	94.7 ± 16.2	97.7 ± 13.2	102.1 ± 12.2	<0.001	620
<92	134 (21.6)	14 (46.7)	18 (37.5)	102 (18.8)	<0.001	620
⊿GNRI	6.8 ± 8.2	-1.7 ± 6.5	3.2 ± 6.6	7.5 ± 8.1	<0.001	572

Values are number (%), mean ± standard deviation (SD), or median (interquartile range). P values were calculated using the chi square test for categorical variables, and 1-way ANOVA or Kruskal-Wallis test for continuous variables. The changes (delta, Δ) were calculated according to the following equation: (the value at 6-month visit) - (the value at discharge).

ACEI, angiotensin-converting enzyme inhibitor; ARB, angiotensin-receptor blocker; BMI, body mass index; BP, blood pressure; BNP, brain-type natriuretic peptide; eGFR, estimated glomerular filtration rate; GNRI, geriatric nutritional risk index; HFrEF, heart failure with reduced ejection fraction; LVEF, left ventricular ejection fraction; MRA, mineralocorticoid receptor antagonist; NT-proBNP, N-terminal pro-brain-type natriuretic peptide; NYHA, New York Heart Association.

* Risk-adjusting variables selected for the Cox proportional hazard model.
