# Supplementary material for: Weight loss during follow-up in patients with acute heart failure: From the KCHF registry
Source: PLoS One. 2023 Jun 23;18(6):e0287637. doi: 10.1371/journal.pone.0287637 (PMC10289349; doi:10.1371/journal.pone.0287637)
Supplement: Attachment — Submitted filename: Response BW R1 seko 0514.docx [file pone.0287637.s010.docx]

***Response***

***We thank the reviewers for careful assessment and positive comments. The manuscript has been rechecked and the necessary changes have been made in accordance with the editors’ and reviewers’ suggestions.***

Reviewer #1: In this prospective observational study, the authors analyzed the correlates and prognostic implications of weight loss at 6-month follow-up in patients discharged after an episode of AHF. Interestingly, weight loss was associated with worse clinical phenotype and outcomes.

The authors should be congratulated for the original idea and the design of the study.

Some points should be addressed to improve the quality of the work:

- How was the 5% cut-off selected? Please detail.

***Response***

***Thank you for your valuable comment. We have selected 5% cut-off because cachexia is primarily defined as a loss of body mass of more than 5% in 12 months and previous studies also have selected 5% cut-off. (Reference [4, 6]) We have included some references in method section. (Page 8, line 12)***

- Beyond the pre-specified cut-off, was weight loss linearly related to worse outcome? A spline curve could express this graphically.

***Response***

***Thank you for your careful assessment and suggestions. A spline curve could express weight loss linearly related to worse outcome graphically, but it is difficult to be used in this study in terms of low patient numbers. We have performed additionally sensitivity analysis classified into three groups as follows: ≥10% decrease in body weight, -10%< body weight change ≤ -5% and no weight loss (reference, -5%< body weight change) (S2 Fig). Higher decrease in body weight was related to worse outcome.***

- No difference was reported in NT-proBNP levels at follow-up visit, despite the well-known correlation with body composition (e.g., 10.1016/j.jchf.2021.05.014). Please discuss.

***Response***

***Thank you for your careful assessment. We have included above reference and discussion as follows: “In our study, no difference was reported in NT-proBNP levels at follow-up visit due to the small number of patients with data and large variability.”(Page 19, line 10-12)***

- It has been reported that the interaction between body composition and outcomes in HF patients may depend on HF etiology (i.e., ischemic vs. non-ischemic, 10.1177/2047487320927610). Please discuss.

***Response***

***Thank you for your careful assessment. Gentile et al. reported that higher BMI were associated with better outcome in non-ischaemic heart failure, but not in ischaemic***

***heart failure. (Eur J Prev Cardiol. 2021;28(9):948-55.)***

***In our study, we focus on weight loss, but we have evaluated the interactions between the ischemic etiology and the effects of weight loss relative to no weight loss on all-cause death.***

***The ratio of ischemic etiology was not significant difference between weight loss and no weight loss group (Table 1). In addition, there was not interaction*** ***between weight loss and outcomes may depend on HF etiology in subgroup analysis (S5 Fig).***

- The lack of physical training is a well-known determinant of both weight loss and poor outcome in HF patients. Did any patient undergo cardiac rehabilitation or training programs after the index hospitalization? In any case this should be matter of discussion.

***Response***

***Thank you for your careful assessment. In this study, we have no data about undergoing cardiac rehabilitation or training programs after the index hospitalization. We have added the next sentence in limitation section. “T******he lack of physical training is a well-known determinant of both weight loss and poor outcome in HF patients, but we have no data about undergoing cardiac rehabilitation or training programs after the index hospitalization.” (Page 20, line 11-13)***

Reviewer #2: The manuscript by Seko and colleagues entitled “Weight Loss During Follow-up in Patients with Acute Heart Failure: from the KCHF registry” is very interesting.

The management of weight in patients with heart failure is a huge problem. In general, weight gain suggests worsening of heart failure, and physicians and patients often aim to maintain or reduce body weight. On the other hand, this paper is highly significant in that it shows that weight loss does not necessarily lead to a favorable prognosis, as pointed out in this paper.

However, I have some comments.

Major comments

1.In patients with heart failure, weight loss is not all bad, and there appears to be malignant weight loss as shown in this paper and benign weight loss in patients who need “intended” reduction of body weight. Therefore, I think, if possible, it would be better to add subgroup analyzes such as baseline body weight-specific analysis.

***Response***

***Thank you for your careful assessment and valuable comments. We have evaluated the interactions between the subgroup factors such as age, etiology of HF, LVEF, BMI, albumin, anemia, geriatric nutritional risk index (GNRI) and the effects of weight loss relative to no weight loss on all-cause death after 6-month visit.*** ***There was no significant interaction between the effect of weight loss relative to no weight loss on the primary outcome measure and the subgroup factors (S5 Fig).***

***We have includes the next sentences as follows: “In addition, there was a trend that patients with low BMI, low albumin levels, or low GNRI at 6-month visit were lily to have more risk when compared to those without, although there was no significant interaction between the effect of weight loss relative to no weight loss on the primary outcome measure and the subgroup factors.” (Page 19, line 18-22)***

2.To prevent weight loss in patients with heart failure, nutrition management and maintenance of activity are important, as the authors also stated. If possible, a more detailed baseline assessment of nutritional status (such as geriatric nutritional risk index) should be added. Among them, if the clinical characteristics of the group that loses weight in a group with maintained nutritional status is clarified, the target of intervention will be clarified a little more.

***Response***

***Thank you for your careful assessment and valuable comments. We have added the data of geriatric nutritional risk index and its change in Table 1. GNRI was lower and the increase of GNRI was less in the weight loss group (Table 1). We have included the next sentences as follows: “The increase of GNRI was less in the BW loss group. These data suggest the importance of nutrition management and maintenance of activity during the follow-up for patients with AHF.” (Page 19, line 16-18)***

3.Why is the KM curve for all-cause death at 30 days getting different? I think factors other than weight loss are important.

***Response***

***Thank you for your careful assessment and valuable comments.*** ***It is not clear why the KM curves for all-cause mortality at 30 days differ. There are two suggested reasons for this: 1) the number of patients enrolled in this study is relatively small and the KM curves for the two cohorts are not smoothed; 2) it is possible that some of the patients who lost weight after discharge may have further improved congestion, making it difficult for them to have events immediately after the starting point of 6 months after discharge. We have added into the Discussion sections (Page 18, line 11-15)***

Minor comment

It may be problematic that about 17% of all patients were analyzed.

***Response***

***Thank you for your careful assessment. We agree with your opinion and described it in the Limitation.***
